# Prospective multi-centre evaluation of guideline−based artificial intelligence to streamline multidisciplinary tumour board

**DOI:** 10.3389/fonc.2026.1785951

**Published:** 2026-08-19

**Authors:** Oleksandr Ivashchuk, Serhiy Hovornyan

**Affiliations:** Department of Oncology and Radiology, Bukovinian State Medical University, Chernivtsi, Ukraine

**Keywords:** artificial intelligence, cancer, cancer diagnostics, cancer treatment, guideline, LLM, machine learning, tumour board

## Abstract

**Introduction:**

Multidisciplinary tumour board (MTB) or tumour board (TB) is the “gold standard” for providing oncological patients’ diagnosis and treatment. Often, MTBs are time-intensive, capacity-constrained, and absent or inconsistent in many routine hospitals. Despite that, in MTB, a few experienced specialists in the oncological field, sometimes the final decision is mistaken (4-47%), the necessity of treatment plan changing (12-64%), etc. Using artificial intelligence (AI) for cancer patients’ treatment is a modern approach, but the main idea of our investigation should be to possibly replace MTB. On the other hand, the choice of correct treatment in oncological patients for some doctors, like surgeons, gynaecologists, and family medicine doctors, is a big trouble. They need to address the guidelines, which are so complicated quick changing. We have offered our AI algorithm, which connects doctors with patients’ data, guidelines (only official sources), and then uses LLM for decision-making. Main research question – assess the role of our AI algorithm in diagnosis and treatment for cancer patients, compare with or together with MTB.

**Methods:**

Prospective, multicentre study at four specialised regional oncological hospitals. Trail period 2023-2025. Additionally, we included 37 doctors (DR) from general and rural hospitals (15 surgeons, 11 gynaecologists, 11 family medicine doctors). 728 cases in eight tumour groups were included in the study: breast 18.13% (132), colorectal 22.12% (161), lung 30.08% (219), prostate 8.79% (64), gynaecological 8.1% (59), gastric 6.59% (48), urothelial 3.99% (29), head & neck 2.2% (16). They were divided into 4 groups: 1) Diagnosis (D) and treatment plan (TP) with MTB only - 226 patients, 2) D and TP with AI only - 206 patients, 3) D and TP with MTB + AI - 147 patients, 4) D and TP with DR + AI - 149 patients. NCCN guidelines were used for all groups. Patients’ data were ingested from the e-health system, de-identified, and normalized prior to inference. Evaluation criteria. Immediate - preparation time (PT), decision time (DT), time to recommendation (TTR), where TTR = PT + DT; Midterm - change diagnosis, change treatment plan (1 month, 6 months). We have collected MTB reports and for group N4 - questionnaires.

**Results:**

Our proposed AI algorithm works as an intermediary between the patient, the doctor, MTB, guidelines, and LLMs. It processes data at each stage for structuring, unification, conversion, machine-readable representation, creates appropriate Python classes, and ultimately a structured supervisory layer with a decision threshold 80%. For immediate criteria we have next results (min): group 1 - PT – 21.3 ± 3.6; DT – 15.6 ± 6.2; TTR – 36.9 ± 7.8; group 2 - PT – 19.7 ± 4.1; DT – 2.1 ± 0.02; TTR – 22.4 ± 3.3; group 3 - PT – 26.9 ± 4.2; DT – 4.9 ± 0.9; TTR – 29.2 ± 3.1; for midterm criteria (change D) results were: on 1 month – group 1 (6.79%), group 2 (5.36%), group 3 (2.78%); on 6 month – group 1 (18.22%), group 2 (19.8%), group 3 (12.4%); midterm criteria (change TP) results were: on 1 month – group 1 (11.31%), group 2 (8.78%), group 3 (4.17%); on 6 month – group 1 (25.12%), group 2 (22.84%), group 3 (15.5%); midterm criteria (change D + TP) results were: on 1 month – group 1 (13.12%), group 2 (8.78%), group 3 (4.86%); on 6 month – group 1 (28.08%), group 2 (24.3%), group 3 (14.73%). The combination of the MTB with AI gives the most effective result in reducing the change in D and TP from 28.08% to 14.73% within 6 months. For group 4, we have found next results. Before the implementation of AI, the time for TP decision in 78.4% of cases was from 30 to 60 minutes, and in 21.6% - more than 60 minutes. At 6 months, DR has time for TP decision, no more than 30 minutes in 67.7%, and among 30 and 60 minutes, 32.3%. Overall assessment using AI for TP in 3 months (good and very good) – 82.8%, in 6 months – 97.1%. Convenience AI in 3 months (good and very good) – 54.2%, in 6 months – 94.1%. Use AI after the end of the study in 3 months (good and very good) – 54.3%, in 6 months – 54.7%. Use in clinical practice the combination of MTB and AI, optimize TTR, decrease the percentage of change D and TP in 1,6 months, decrease the negative sides of single MTB and AI. We can use this combination for first-detected cases without comorbidities at a basic level. AI cannot replace MTB, but it empowers non-oncological clinicians and DR to generate evidence-based, auditable recommendations in settings with limited specialist capacity.

**Discussion:**

The decrease in the percentage of change D and TP after MTB using AI in the first detected cases highlights the importance of conducting further studies to optimize MTB performance. The positive results of using DR assistance in the form of AI indicate the need to create other practical tools, such as an app, a pocket version, etc. To reach expert-level autonomy, further work is required: a local guideline adaptation, multimodal data ingestion, and longitudinal validation across tumour types, tumour recurrence, multiple cancers, comorbidity, and assessment of the financial efficiency of implementing AI in the work of the MTB.

## 1 Introduction

Oncology is one of the most complicated fields in modern science of human health. Multidisciplinary tumour board (MTB) (in different countries, it has another name) is the best solution for correct diagnosis (D) and treatment plan (TP) for cancer patients. In MTB, experienced oncologists, oncogynaecologists, breast cancer specialists, radiologists, chemotherapists, pathomorphologists, etc., are included. In spite of that, there are many problems connected with correct D and TP (1–3). Let’s go into more detail.

First of all, incomplete or incorrect data in the patient cards during preparation for the MTB meeting (lack of images, histology, CT/MRI/PET data), different interpretation of images/pathology between specialists, differences in local protocols or resources (regional restrictions on access to therapies/technologies), patient preferences or clinical management steps after the meeting that change the implementation of the recommendation, low quality of evidence for certain clinical situations - leads to variability in recommendations (4, 5).

Secondly, large variability between centres and countries - discrepancies in diagnosis/staging and recommendations are often related to organizational differences, diagnostic availability, data availability, and local protocols. Recent studies show that discordance in recommendations can vary widely depending on the study design and type of comparison, from a few percent to ~50% in narrow comparisons (depending on criteria and quality of evidence) (6, 7).

Thirdly, some criteria for correct assessment in trials, which concern MTB efficacy - different definitions of “change” (what is considered significant) and different protocols for recording changes, different study designs (prospective vs retrospective; single-country vs multi-country), differences in case reporting practices (data completeness, availability of images and pathology), and tumour type. Unfortunately, we don’t have a universal approach for MTB assessment and comparison (4, 8, 9).

Today, the need to change the D or TP occurs in 4 to 67% of cases at different times after MTB (depending on different cancer types) in modern oncological centres. This leads to complications, reduced treatment effectiveness, deterioration in the patient’s quality of life, prolonged treatment duration, and an additional financial burden (10, 11).

On the other hand, if the issue of determining D and TP is assigned to an ordinary doctor (surgeon, gynaecologist, family doctor, thoracic surgeon, urologist, etc.), then the results here are significantly worse compared to MTB. This is due to the fact that for this doctor, oncological patients are one of many others that they deal with. It is much more difficult for them to follow clinical recommendations and their updates, and to treat cancer in certain localizations (11, 12).

How can we increase results for MTB and other doctors, who are responsible for cancer diagnosis and treatment? The most important approach that we can offer is to try to use AI algorithms. Nowadays, AI is widely involved in different areas in oncology: diagnosis, treatment, developing new drugs, etc. It means that AI is rapidly entering clinical oncology as a tool for clinical delivery support (CDS), personalization of therapy (based on images, molecular profiles, and genetic features), patient selection for clinical trials, and prediction of response/outcome. Preliminary reviews and systematic reviews from 2024–2025 show an increase in both publications and early clinical applications, but at the same time, there are notable challenges with validation, interpretability, and regulatory issues (13, 14).

Main areas of application of AI in determining treatment tactics: clinical decision support systems — ranking of therapeutic options, assistance in interpreting molecular tests, recommendations on targeted drugs/immunotherapy (regulatory documents determine when such software is a medical device); radiomics and digital pathology — DL-models extract predictive biomarkers from CT/MR/PET and pathomorphological sections to predict response to chemo/radio/immunotherapy; genomics/omics approaches — models based on tumour sequencing (WES/RNA-seq) for selection of targeted therapies or prediction of sensitivity; Cancer Genome Atlas is a key resource for training models; clinical trial recruitment and protocol optimization — algorithms find suitable patients and predict responses/risks. There are examples of implemented projects and initial research (15, 16).

Among the known, the most common are the following methods and architectures: classical machine learning - logistic regression, random forests, XGBoost — often for tabular clinical data; deep learning - CNN for images, transformers and integrative models for multi-omic data; federated learning and synthetic data — for privacy and sample expansion. An important detail: models that work well on retrospective sets often generalize poorly without external validation (17–19).

Limitations and risks: insufficient external validation and replication of results; difference between correlation and causation - the model can predict, but not substantiate, the mechanism of action of the therapy; risk of overfitting, especially with high-dimensional omics data and small cohorts (20, 21).

Evidence base and validation for AI: mostly retrospective studies and internal cross-validation, there is a growing number of prospective trials and clinical studies, but randomized controlled trials are still limited; regulators (FDA) are issuing guidance for the use of AI in clinical and regulatory decisions; for clinical decision support systems, there are documents that delineate when software is regulated as a medical device; this is critical when developing systems that advise on therapy (17, 22).

Ethical, legal and practical issues that are important when using AI in oncology should not be forgotten: interpretability and clinician trust - “black boxes” reduce adoption, explanatory mechanisms (explainable AI) are needed; bias and inequalities - data containing predominantly patients from a single geography/ethnicity yield biased predictions; liability for erroneous recommendations - an unresolved issue in many jurisdictions; data privacy and security - the need for federated approaches and thorough de-identification (17–19, 23).

Another thing that AI developers for oncology encounter is the widespread use of anthropomorphism among doctors. Anthropomorphism – the integration of artificial AI into oncology has coincided with an expanding use of human-centred terminology. Such anthropomorphism - when algorithms are described as if they possessed cognition, intent, or moral agency - risks distorting conceptual clarity and weakening scientific rigor. Globally, oncological AI literature exhibits a gradual shift toward linguistic precision, privileging technical over metaphorical framing and reflecting a maturing scientific discourse. Reducing reliance on anthropomorphic language may enhance conceptual rigor, prevent diffusion of responsibility, and support more accountable and balanced discourse in AI-driven oncology research (24, 25).

Thus, to date, there is no correct AI tool to address current issues in clinical oncology. Research needs to develop new AI algorithms that would satisfy both highly specialised and non-specialised physicians.

## 2 Materials and methods

### 2.1 Study design

For receiving correct results, we have used a prospective, multi-centre study at four specialised regional oncological hospitals, where experienced MTB functioning (consisting of an oncosurgeon, oncogynaecologist, breast cancer specialist, pathologist, radiologist, radiotherapy specialist, chemotherapy specialist, secretary, technical assistant, and others as needed). All specialists in MTB had at least 15 years of experience in their field. For analysis, we collected MTB reports during the period 01.01.2023-01.10.2025. Additionally, we included 37 doctors (DR) from general and rural hospitals (15 surgeons, 11 gynaecologists, 11 family medicine doctors), who are involved in the diagnosis and treatment of cancer patients. They filled out questionnaires in Google Forms. Before starting the study, we had provided 2 hours of training webinars or live lessons on how to use the AI algorithm and collect data for study participants. The training period was 1 month.

728 cases in eight tumour groups were included in the study: breast 18.13% (132), colorectal 22.12% (161), lung 30.08% (219), prostate 8.79% (64), gynaecological 8.1% (59), gastric 6.59% (48), urothelial 3.99% (29), head & neck 2.2% (16). Inclusion criteria: age 20–60 years (37,6 ± 8.3), regardless of gender, first detected cases. Exclusion criteria: moderate and severe concomitant disease, previously treated cancer, relapse of cancer, and patient (or his family) doubts about the use of AI.

**Figure 1:**
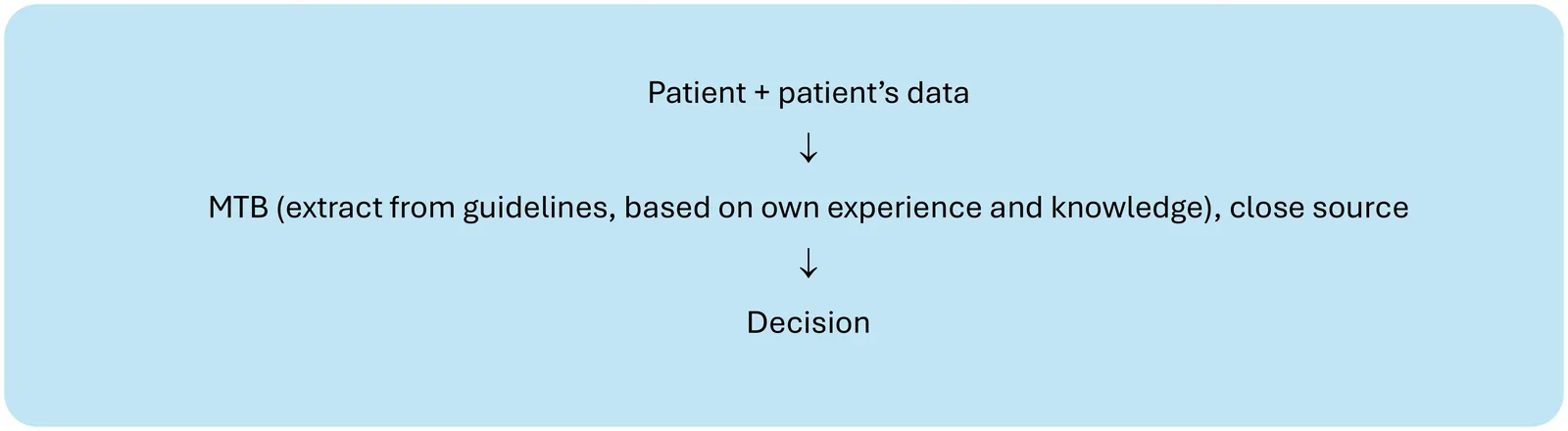
D and TP decision in 1st group algorithm. It means, that MTB analyze whole patient's information, including assessment of his condition. After discussion, MTB makes decision, which based on own experience and presented data.

**Figure 2:**
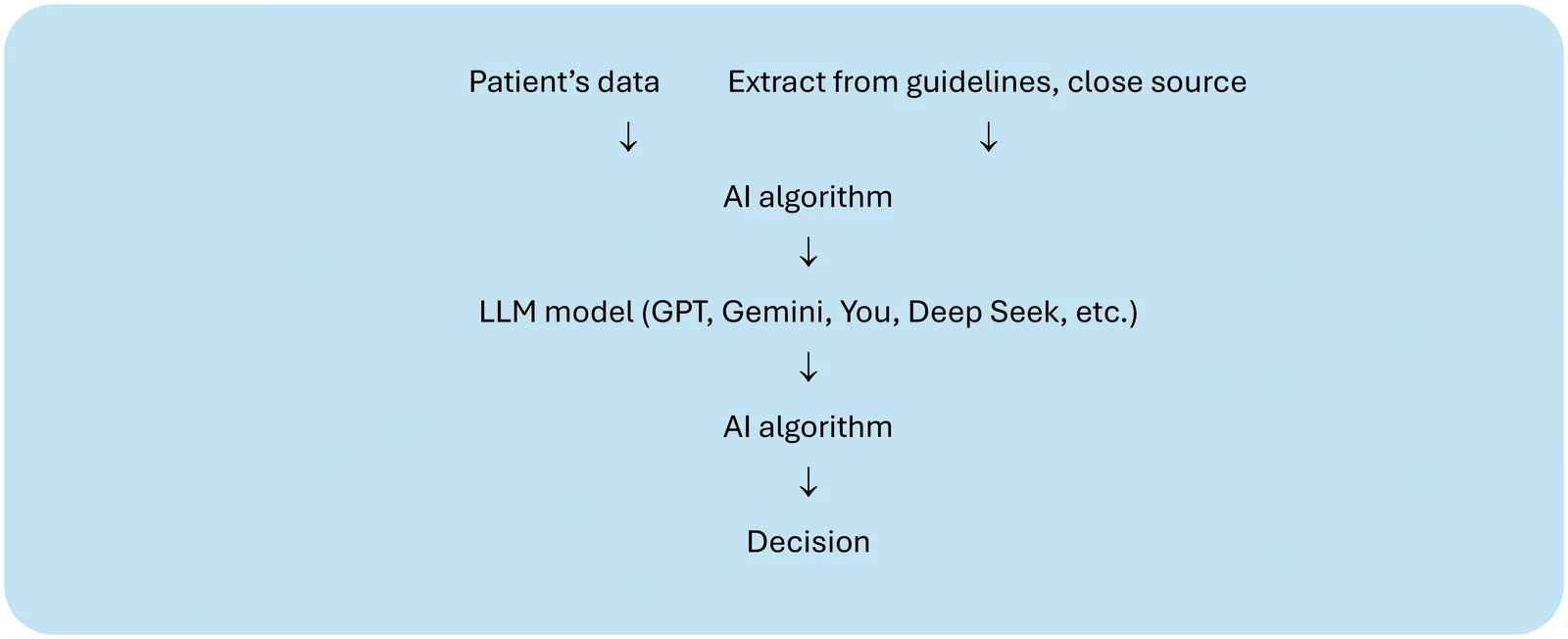
D and TP decision in 2nd group algorithm. In this case, Al takes only patient's data, downloaded guidelines and address directly to external LLM model, which gives decision (based on all information and questions).

**Figure 3:**
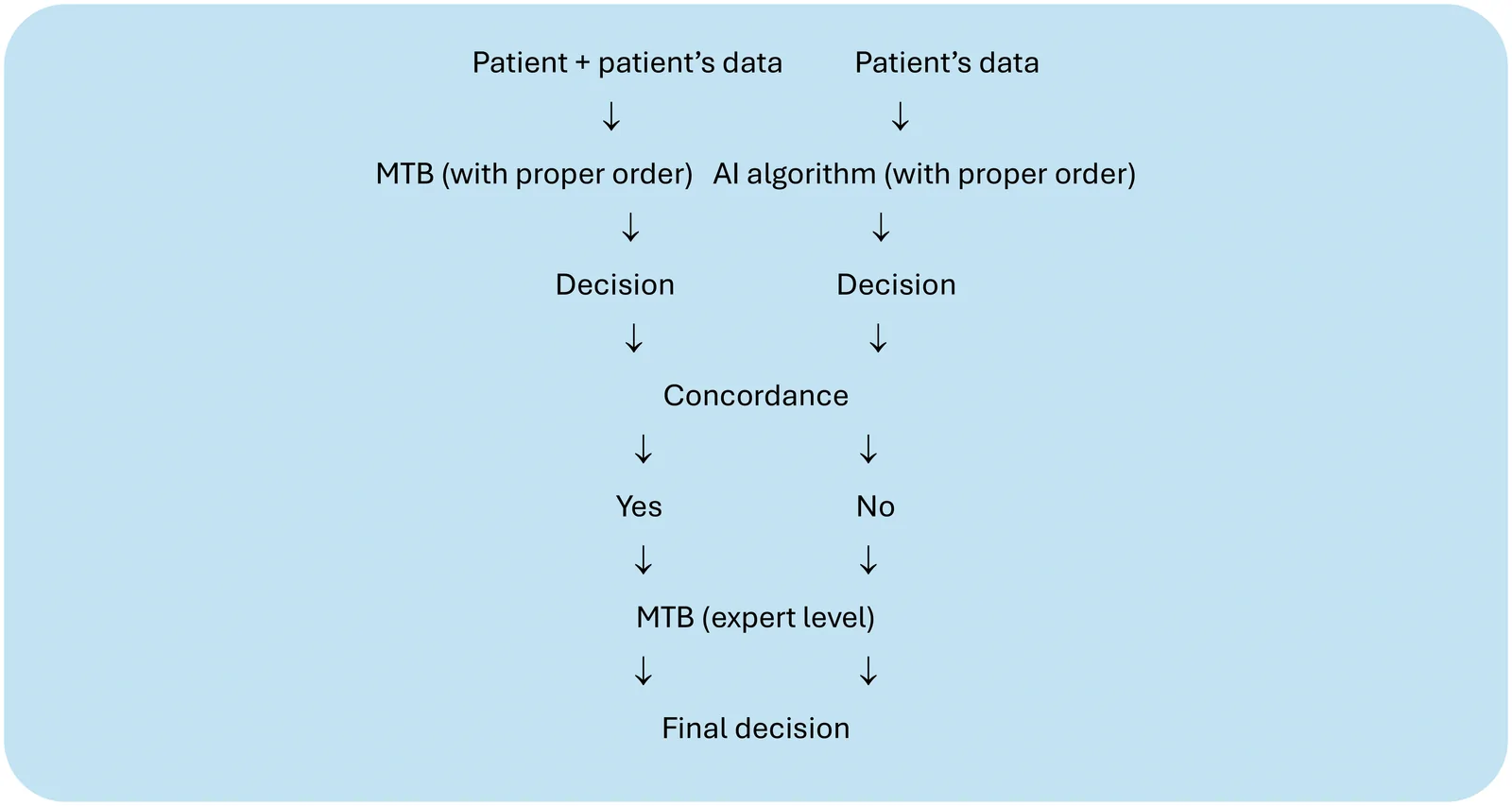
D and TP decision in 3rd group algorithm. In 3 group we unite paths of 1 and 2 group. Main difference, that in case of non-concordance MTB and Al decision, we must attract MTB again for final decision.

**Figure 4:**
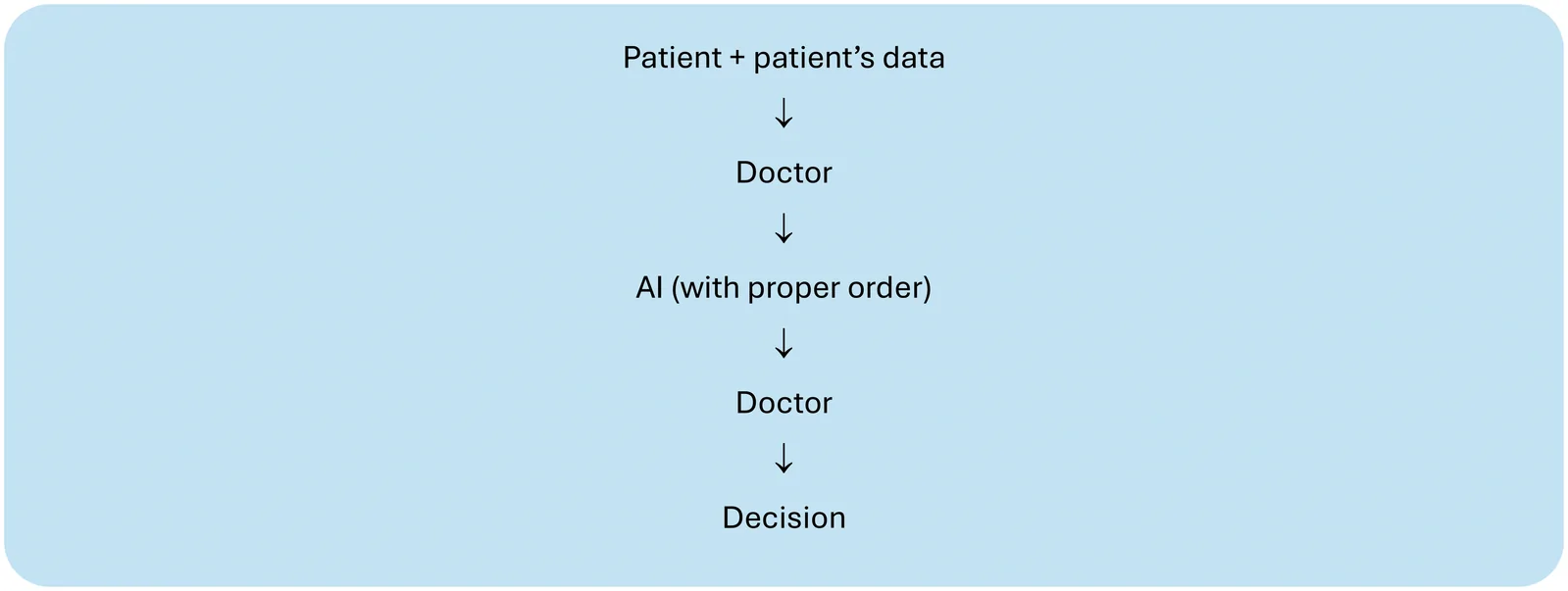
D and TP decision in 4th group algorithm. Algorithm for use by single doctor same to group 2, but here only doctor makes decision.

They were divided into 4 groups:

1. D and TP with MTB only - 226 patients (the algorithm is presented in **Figure 1**)
2. D and TP with AI only - 206 patients (the algorithm is presented in **Figure 2**)
3. D and TP with MTB + AI - 147 patients (the algorithm is presented in **Figure 3**)
4. D and TP with DR + AI - 149 patients (the algorithm is presented in **Figure 4**)

NCCN guidelines were used for all groups. Patients’ data were ingested from the e-health system, de-identified, and normalized prior to inference. All patients included in the study signed informed consent. Also, the local bioethics committee was permitted to study.

Reports from MTB were inserted in the proper Google Forms.

For effectiveness assessment, we used evaluation criteria: immediate (better understanding of how AI can influence diagnosis) - preparation time (PT), decision time (DT), time to recommendation (TTR), where TTR = PT + DT; midterm (for correct results after MTB, AI, and both decisions) - change diagnosis, change treatment plan (1 month, 6 month).

Questionnaire for DR includes next questions: overall assessment using AI for D, overall assessment using AI for TP, convenience AI, ease of use AI, use AI decreases the time for D and TP decisions, recommendation to colleagues for AI use, and use AI after the end of the study. Answers were (on 3rd and 6th month of study): 0 – very bad or strictly negative, 1 - bad or negative, 2 - moderate or not sure, 3 – good or positive, 4 - very good or strictly positive.

### 2.2 Statistical analysis

Descriptive analyses for ordinally scaled data (e.g., rankings or numerical scores) used mean, standard deviation (SD), median, and 95% confidence intervals (CI). Changes versus baseline were either expressed in absolute or relative terms for scores as well as proportions. Significance was set at p<0.05 (two-sided).

## 3 Results

### 3.1 Technical aspects of our own AI algorithm implementation

Our AI algorithm was created with one important reason – to help decrease errors for MTB and any doctor in their routine work with D and TP for cancer patients. It can be provided by building an AI algorithm that unites all the needed parts.

The hierarchy of our AI includes many components, but strategically we can distinguish three main stages that are performed sequentially.

#### 3.1.1 Introduction to the three-stage AI decision pipeline

The proposed AI algorithm operates within a decision-support workflow designed to approximate the reasoning of a multidisciplinary tumour board while remaining technically tractable, auditable, and modular. Clinicians interact with the system through a web interface by providing three heterogeneous inputs: clinical practice guidelines (as PDF documents), patient-specific data (in structured form), and free-text clinical questions. These inputs differ substantially in structure, reliability, and semantic complexity, and cannot be processed safely as a single undifferentiated stream. To manage this complexity, the AI workflow is explicitly decomposed into three sequential stages.

The first reason for this staging is the separation of concerns. Converting noisy, human-facing artefacts into machine-readable representations is a fundamentally different task from generating recommendations, and both are distinct from validating those recommendations against safety and concordance criteria. By isolating these tasks, each stage can use specialised prompting strategies, external language models, and rule-based logic optimised for its role, while communicating via stable, typed Python classes(e.g., GuidelineRepresentation, PatientClinicalProfile, ParsedRequest). This improves maintainability and allows individual components to be updated or replaced without destabilising the overall system.

A second reason is traceability and governance. The staged design forces the algorithm to expose intermediate representations and decisions instead of collapsing all reasoning into a single opaque model call. This enables explicit inspection of what the system has “understood” about the guideline, the patient, and the clinician’s request before any recommendation is issued. It also creates natural checkpoints for logging, error detection, and performance evaluation at each stage.

Finally, the third reason is safety and calibration. Preliminary decisions produced from structured inputs are not delivered directly to clinicians; they must pass through a dedicated supervisory stage that assesses concordance with guideline logic and patient-specific constraints. This separation between decision formation and decision validation is essential in a clinical research context, where the system must support, rather than replace, expert judgement and where failures must be detectable, explainable, and correctable.

#### 3.1.2 AI algorithm Stage 1: ingestion of inputs and conversion to machine-readable format

**Figure 5:**
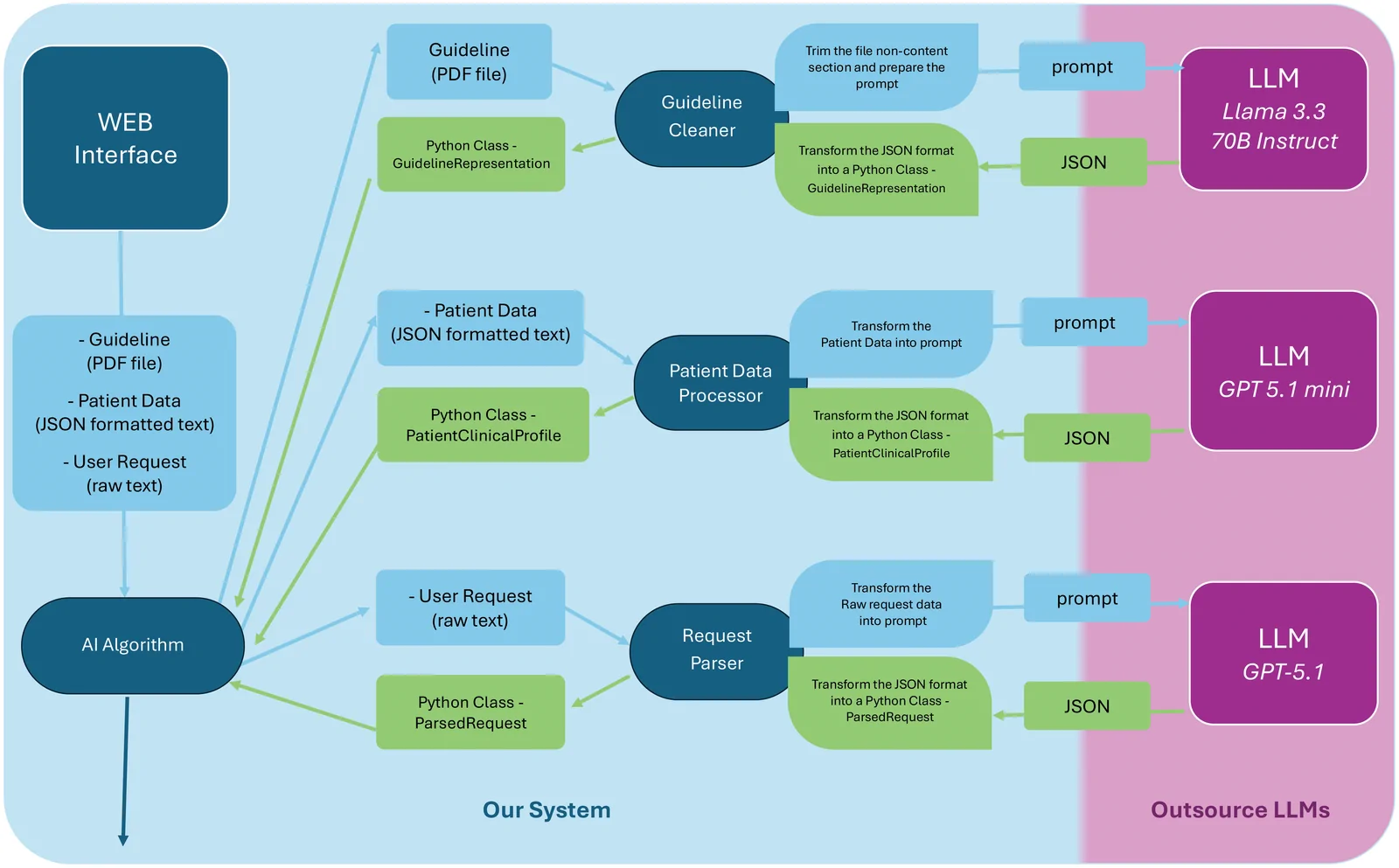
Al algorithm stage 1: ingestion of inputs and conversion to machine-readable format.

In Stage 1, the AI algorithm transforms three heterogeneous input streams into a unified, machine-readable representation that can be consumed by downstream components. The system receives: a guideline document as a PDF file, patient data as JSON-formatted text, and the user request as unstructured natural language. Each of these inputs is routed to a dedicated module—Guideline Cleaner, Patient Data Processor, and Request Parser, respectively—whose primary function is to interface with outsourced large language models (LLMs), enforce structured output, and encapsulate the result in typed Python classes (GuidelineRepresentation, PatientClinicalProfile, and ParsedRequest) (**Figure 5**).

The Guideline Cleaner module operates on the guideline PDF, which is first converted into raw text using standard document parsing tools. The extracted text is segmented into logical units (sections, paragraphs, tables) that are then formatted into prompts for an external LLM. These prompts instruct the model to identify and extract atomic clinical recommendations, eligibility criteria (e.g. tumour site, TNM stage, performance status constraints), and levels of evidence, and to return the results in a predefined JSON schema. The system enforces this schema by rejecting or reparsing non-conforming outputs and, where necessary, applying *post-hoc* rule-based corrections. The validated JSON is then deserialised into the GuidelineRepresentation Python class, which captures both structured recommendations and references to the underlying text segments for subsequent use.

The Patient Data Processor ingests patient data supplied as JSON from the user-facing interface. Although structurally formatted, this input typically reflects user-interface and local documentation conventions rather than a canonical oncologic representation. The module, therefore, constructs prompts that ask an outsourced LLM to normalise the data onto a domain-specific schema, including primary diagnosis (tumour site, histology, TNM, and stage group), performance status, comorbidities, prior treatments, and salient laboratory or functional parameters. The LLM returns a JSON object that is constrained to a predefined structure; deterministic rules are then applied to derive secondary attributes such as frailty status, treatment contraindications (e.g., cisplatin ineligibility), and other clinically relevant flags. The final, validated representation is stored in the PatientClinicalProfile class, which serves as the canonical “snapshot” of the patient used throughout subsequent stages.

The Request Parser processes the clinician’s free-text question. Its goal is to convert a natural-language request (e.g., “What is the recommended primary treatment for this T2N1M0 oral cancer in a cachectic patient?”) into a formal query specification. The module constructs prompts that instruct the outsourced LLM to infer the task type (diagnostic, treatment, follow-up), tumour site, stage or stage hypotheses, treatment intent, and explicit constraints or preferences stated in the question. The LLM is required to respond using a strict JSON schema, from which the system instantiates the ParsedRequest class. This object encodes what the system believes is being asked, independent of the particular wording, and includes uncertainty flags if key elements cannot be reliably inferred.

Across all three modules, a common pattern is applied: inputs are decomposed into prompts targeting outsourced LLMs, expected outputs are defined as JSON schemas, and any deviations from these schemas are captured through validation and error-handling routines. The resulting Python classes—GuidelineRepresentation, PatientClinicalProfile, and ParsedRequest—constitute the complete machine-readable state at the end of Stage 1. This staged conversion isolates the noisy, human-facing aspects of the problem in a single phase, ensuring that subsequent stages operate exclusively on well-typed, semantically aligned artefacts rather than raw text or *ad hoc* data structures.

#### 3.1.3 AI algorithm Stage 2: structured prompt generation and preliminary decision formation

**Figure 6:**
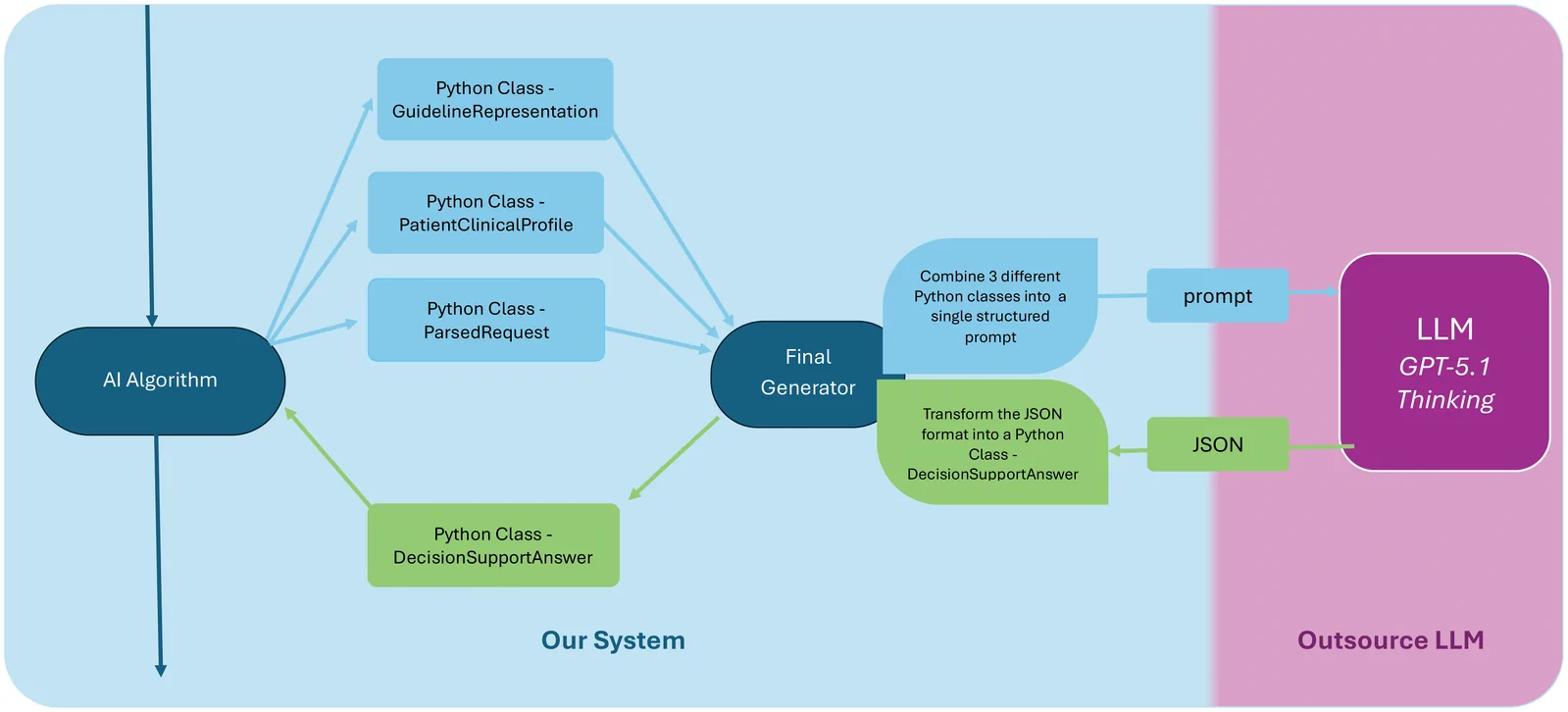
Al algorithm stage 2: structured prompt generation and preliminary decision formation.

Stage 2 processes the core decision-making step of the AI algorithm by transforming the structured representations from Stage 1 into a preliminary recommendation. At this point, all human-facing heterogeneity has been abstracted into three typed Python classes: GuidelineRepresentation, PatientClinicalProfile, and ParsedRequest. The Final Generator module is responsible for integrating these artefacts into a single structured prompt, submitting this prompt to an outsourced large language model (GPT-5.1 Thinking), and translating the model’s response into an internal decision object (DecisionSupportAnswer) (**Figure 6**).

The process begins with context assembly. The Final Generator receives instances of GuidelineRepresentation, PatientClinicalProfile, and ParsedRequest and performs a selective transformation into a compact prompt payload. From the guideline representation, it extracts only those recommendations and text segments whose eligibility criteria match the patient’s stage, tumour site, and key constraints as encoded in the patient profile. From the patient profile, it constructs a succinct summary of the diagnosis (site, TNM, stage group), performance status, comorbidities, prior treatments, and derived flags (e.g., frailty, treatment contraindications). From the parsed request, it retrieves the task type, treatment intent, and any explicitly stated constraints or user preferences.

These elements are then assembled into a highly structured system prompt that specifies to GPT-5.1 Thinking both the decision problem and the output contract. The prompt includes: a machine-readable block describing the patient; a block summarising relevant guideline recommendations and evidence annotations; and a block describing the clinical question and required outputs (e.g., primary recommendation, alternatives, non-recommended options, and rationale). Critically, the prompt also embeds a formal JSON schema that defines the expected structure of the model’s response, including fields for the primary recommendation, alternative options, non-recommended strategies with reasons, guideline alignment references, and uncertainties or open issues.

GPT-5.1 Thinking is then invoked as an outsourced reasoning engine, with the Final Generator acting as a strict contract enforcer. The model’s output is accepted only if it conforms to the predefined JSON schema; non-conforming outputs trigger automatic re-prompting or rejection, thereby reducing the risk of malformed responses. Once a valid JSON object is obtained, it is deserialised into the DecisionSupportAnswer Python class. This class encapsulates the preliminary decision, including: a structured description of the primary recommended strategy and its intent; a set of alternative strategies with indications, advantages, and disadvantages; a list of options that are explicitly not recommended for this patient and the reasons for exclusion; and a guideline alignment section that links each proposed option to specific recommendations contained in GuidelineRepresentation.

Stage 2 is deliberately limited to decision formation, not validation. While the Final Generator uses guideline context and patient constraints as conditioning information, it does not independently enforce safety thresholds or concordance metrics. Instead, its role is to produce the best possible candidate answer under full visibility of the structured inputs and to expose its reasoning in a transparent, machine-readable format. The resulting DecisionSupportAnswer object, together with the original guideline and patient representations, is then passed to Stage 3, where a dedicated supervisory process evaluates the consistency, safety, and guideline concordance of the preliminary decision before any output is returned to the user.

#### 3.1.4 AI algorithm Stage 3: supervisor module validation and final decision delivery

**Figure 7:**
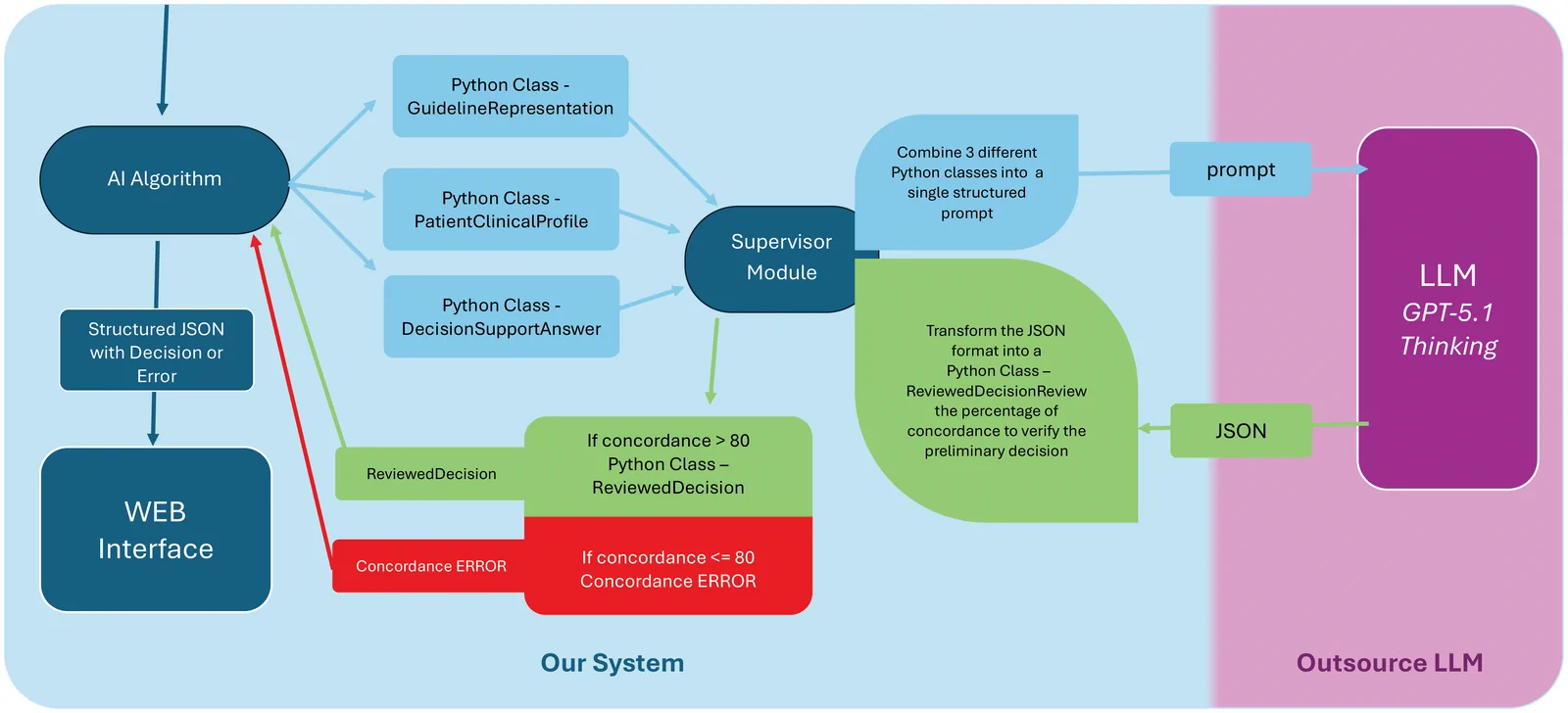
Al algorithm stage 3: supervisor module validation and final decision delivery.

Stage 3 implements a structured supervisory layer that evaluates the preliminary recommendation before it is exposed to the end user. At this point, the AI algorithm has already produced a candidate decision encoded in the DecisionSupportAnswer class and retains full access to the structured representations of guideline knowledge (GuidelineRepresentation) and patient status (PatientClinicalProfile). The Supervisor Module uses these three artefacts to perform an independent consistency and concordance assessment, leveraging an outsourced LLM (GPT-5.1 Thinking) as a critic rather than as a primary decision-maker (**Figure 7**).

The validation process begins with the construction of a supervisory prompt that combines: a compact representation of the patient profile, including diagnosis, stage, performance status, comorbidities, and derived flags; the subset of guideline recommendations relevant to this case, as encoded in GuidelineRepresentation; and the full content of the DecisionSupportAnswer, including primary recommendation, alternatives, non-recommended options, and guideline alignment claims. The prompt explicitly instructs GPT-5.1 Thinking to assess, in a structured manner, whether the proposed decisions are consistent with guideline logic, compatible with patient-specific constraints, and internally coherent.

The LLM is required to output a JSON object conforming to a predefined schema that includes: (a) a concordance score between 0 and 100%, reflecting the degree of alignment between the decision and the guideline–patient combination; (b) a list of identified issues, categorised by severity (e.g. minor omission vs. major contraindication violation); and (c) an optional set of suggested modifications to the decision, including justifications and references to specific guideline elements. The Supervisor Module validates the returned JSON against this schema and, if necessary, re-prompts until a structurally valid response is obtained.

Once a valid supervisory response is available, the algorithm applies a decision threshold. If the concordance score is at or above a predefined value of 80%, the Supervisor Module integrates any suggested minor corrections into the original DecisionSupportAnswer and instantiates a ReviewedDecision class that represents the final, vetted recommendation. This object includes the refined decision, the final concordance score, a summary of modifications relative to the preliminary answer, and a structured list of residual uncertainties that should be highlighted to the clinician.

If the concordance score falls below the threshold, or if the supervisory output flags critical safety issues (e.g., recommendation of a modality that conflicts with a major contraindication encoded in the PatientClinicalProfile), the algorithm does not deliver a recommendation. Instead, it generates an error status that is returned upstream, together with a concise explanation of the detected issues and, where appropriate, suggestions for revising the input (e.g., clarifying missing staging information or correcting inconsistent patient data). In this failure mode, the user is required to revise the case and re-run the algorithm, maintaining a human-in-the-loop model of oversight.

All supervisory interactions, including the concordance score, issue list, and any applied modifications, are logged alongside the underlying inputs and the preliminary decision. This architecture ensures that Stage 3 functions as a transparent, auditable control layer that enforces guideline adherence and patient-specific safety constraints, while still enabling the use of powerful but fallible LLMs as both decision engines and structured critics within a controlled pipeline.

#### 3.1.5 Summary of the staged AI decision pipeline

The proposed three-stage AI pipeline formalises guideline-based decision support as a sequence of well-defined transformations from heterogeneous clinical inputs to a vetted, machine-generated recommendation. In Stage 1, guideline documents, patient data, and clinician queries are ingested and converted into harmonised, domain-specific representations (GuidelineRepresentation, PatientClinicalProfile, ParsedRequest). This isolates the inherently noisy interface between human and machine and ensures that all subsequent reasoning operates on semantically aligned artefacts rather than raw text or *ad hoc* data structures.

Stage 2 leverages these representations to generate a preliminary recommendation via a dedicated Final Generator module. By conditioning GPT-5.1 Thinking on a structured description of the patient, the applicable guideline content, and the formalised clinical question, and by constraining outputs to the DecisionSupportAnswer schema, the system produces decisions that are both context-aware and amenable to downstream analysis.

Stage 3 then introduces an explicit supervisory layer that re-evaluates the preliminary decision against the original guideline and patient profile, using GPT-5.1 Thinking as a structured critic. The Supervisor Module quantifies guideline concordance, identifies safety or consistency issues, and either promotes the decision to a ReviewedDecision or blocks it when predefined thresholds are not met.

Collectively, this staged design combines the expressive power of large language models with principled abstractions, explicit schemas, and threshold-based control. It creates a technically transparent and auditable framework within which AI-generated recommendations can be prospectively evaluated against multidisciplinary tumour board decisions, while preserving a clear role for human oversight and clinical responsibility.

### 3.2 Clinical aspects of our own AI algorithm implementation

There are several key aspects in the work of the MTB that affect the quality of its work - these are urgent and midterm criteria, which served as the subject of our research.

**Figure 8:**
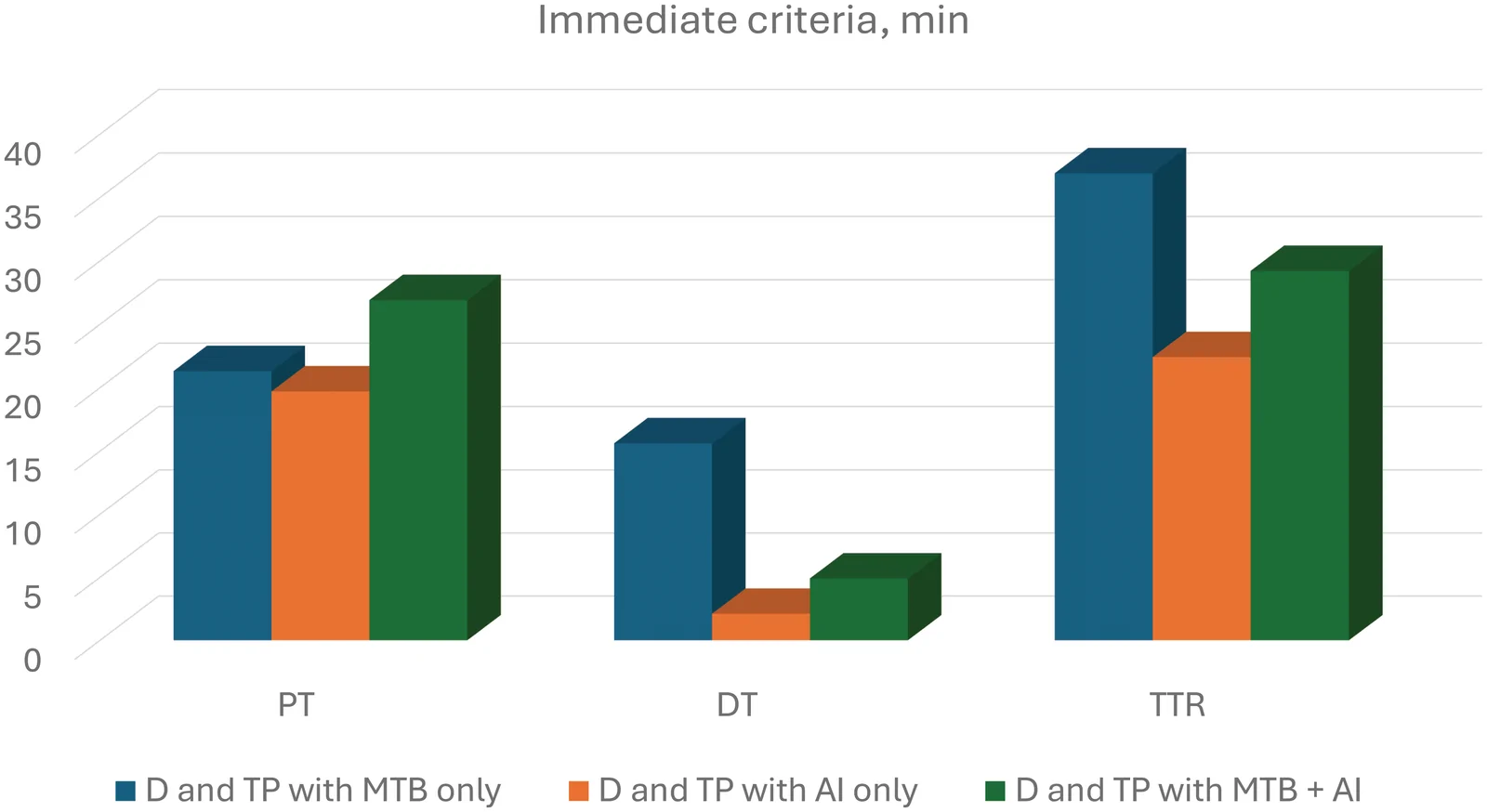
Immediate criteria in different groups.

Assessment begun from immediate criteria, namely time, spending for 1 patient in different groups (**Table 1**; **Figure 8**).

**Table 1:** Immediate criteria (M ± SD), min.

Group	PT	DT	TTR
D and TP with MTB only, n=226	21.3 ± 3.6	15.6 ± 6.2	36.9 ± 7.8
D and TP with AI only, n=206	19.7 ± 4.1	2.1 ± 0.02*	22.4 ± 3.3*
D and TP with MTB + AI, n=147	26.9 ± 4.2*,**	4.9 ± 0.9*,**	29.2 ± 3.1*,**

* statistically significant results in comparison to group - D and TP with MTB only.

** statistically significant results in comparison to group - D and TP with AI only.

The higher PT results in group 3 indicate that more time is needed to load patient data and interpret it accordingly. At the same time, the DT results in group 3 are the lowest, and 7 times less than in group 1.

Group 3 (MTB + AI) has higher scores TTR than in group 2, but still significantly lower than group 1. It means, that adding AI to MTB not only does not extend the TTR, but also reduces it on 21%. The implementation of AI does not prolong the operation of MTB, which allows TTR to be accepted for a larger number of patients per unit of time.

The next stage of our research was to study the impact of AI implementation on midterm criteria, namely whether the implementation of AI in MTB work affected the quality of D and TP, and possible changes in different terms.

**Table 2** presents the results of D change in the terms of patient observation 1 and 6 months after MTB.

**Table 2:** Midterm criteria (change D).

Group	1 month	6 months
D and TP with MTB only, n=226	n^1^ = 221 n^1x^=15 6.79%	n^6^ = 203 n^6x^=37 18.22%
D and TP with AI only, n=206	n^1^ = 205 n^1x^=12 5.36%	n^6^ = 197 n^6x^=39 19.8%
D and TP with MTB + AI, n=147	n^1^ = 144 n^1x^=4 2.78%*,**	n^6^ = 129 n^6x^=16 12.4%*,**

n – number of patients studied on MTB.

n^1^= overall number of patients in 1 month after MTB.

n^1x^= number of patients with change D in 1 month after MTB.

n^6^= overall number of patients in 6 months after MTB.

n^6x^= number of patients with change D in 6 months after MTB.

* statistically significant results in comparison to group - D and TP with MTB only.

** statistically significant results in comparison to group - D and TP with AI only.

**Figure 9:**
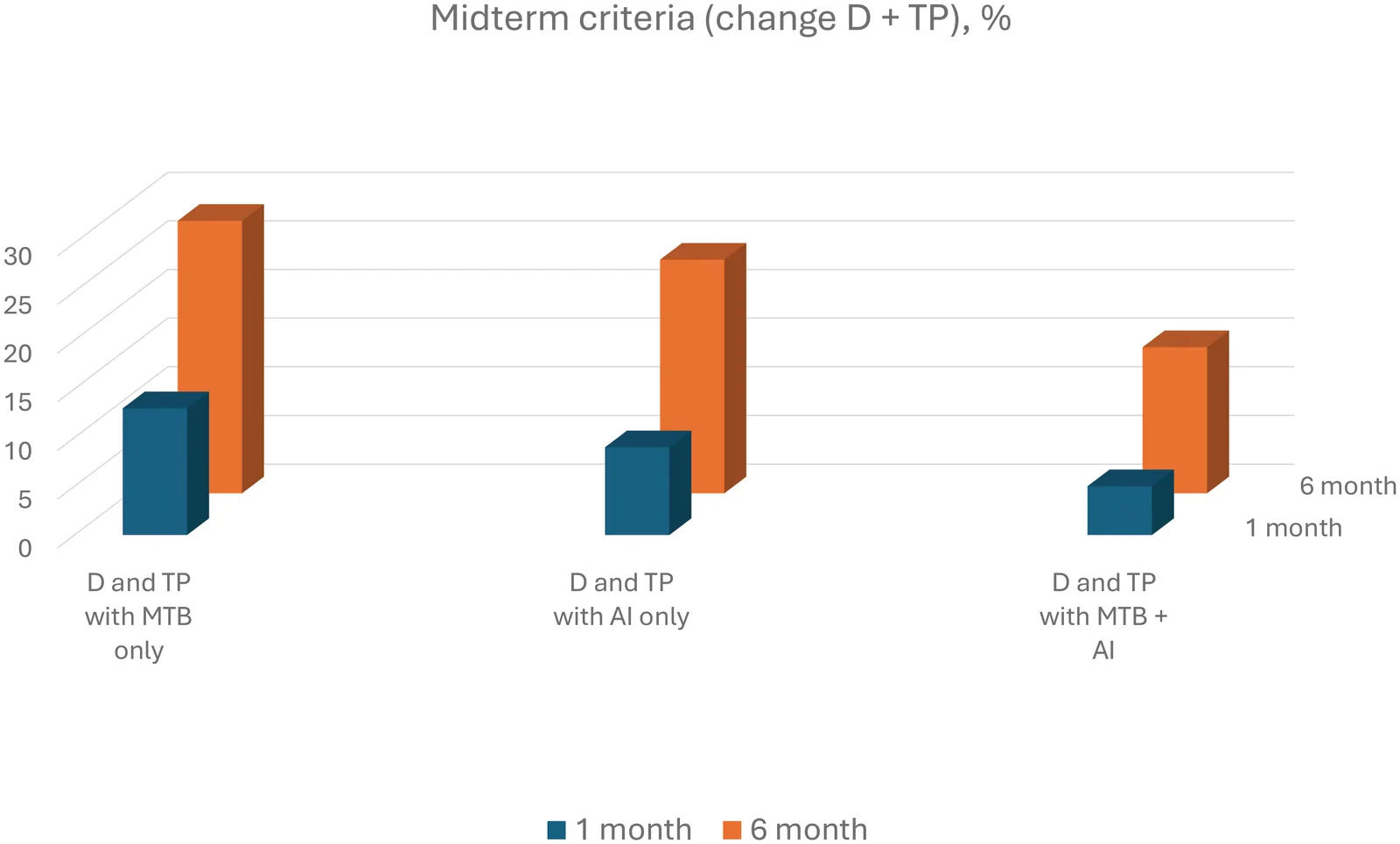
Midterm criteria in different groups.

It should be taken into account that a change in D does not always mean a change in TP, and vice versa, when leaving the previous D, TP should be changed. Therefore, we investigated the role of AI in changing the D and TP (**Tables 3**, **4**; **Figure 9**).

**Table 3:** Midterm criteria (change TP).

Group	1 month	6 months
D and TP with MTB only, n=226	n^1^ = 221 n^1x^=25 11.31%	n^6^ = 203 n^6x^=51 25.12%
D and TP with AI only, n=206	n^1^ = 205 n^1x^=18 8.78%	n^6^ = 197 n^6x^=45 22.84%
D and TP with MTB + AI, n=147	n^1^ = 144 n^1x^=6 4.17%*,**	n^6^ = 129 n^6x^=20 15.5%*,**

n – number of patients studied on MTB.

n^1^= overall number of patients in 1 month after MTB.

n^1x^= number of patients with change TP in 1 month after MTB.

n^6^= overall number of patients in 6 months after MTB.

n^6x^= number of patients with change TP in 6^th^ months after MTB.

* statistically significant results in comparison to group - D and TP with MTB only.

** statistically significant results in comparison to group - D and TP with AI only.

**Table 4:** Midterm criteria (change D + TP).

Group	1 month	6 months
D and TP with MTB only, n=226	n^1^ = 221 n^1x^=29 13.12%	n^6^ = 203 n^6x^=57 28.08%
D and TP with AI only, n=206	n^1^ = 205 n^1x^=18 8.78%*	n^6^ = 197 n^6x^=48 24.3%
D and TP with MTB + AI, n=147	n^1^ = 144 n^1x^=7 4.86%*,**	n^6^ = 129 n^6x^=19 14.73%*,**

n – number of patients studied on MTB,.

n^1^= overall number of patients in 1 month after MTB.

n^1x^= number of patients with change TP in 1 month after MTB.

n^6^= overall number of patients in 6 months after MTB.

n^6x^= number of patients with change TP in 6 months after MTB.

* statistically significant results in comparison to group - D and TP with MTB only.

** statistically significant results in comparison to group - D and TP with AI only.

The generalized assessment shows that the introduction of AI into the work of the MTB significantly reduces the percentage of change in D and TP. At 1 month of the study, the difference between group 3 and 1 was 2.7 times, and at 6 months 1.9 times. The use of AI alone shows better results with TP, but this difference is not statistically significant.

The combination of the MTB with AI gives the most effective result in reducing the change in D and TP from 28.08% to 14.73% within 6 months (13.12% vs 4.86% in 1 month).

A number of excluded patients from study explained by next reasons: refusal to participate in the study, occurrence of exclusion criteria, change of residence.

The next step was to study the effectiveness of using AI in the work of DR (group 4, **Table 5**). The comparison group was taken as indicators of D and TP by doctors before the implementation of AI.

**Table 5:** Technical aspects of AI use for DR.

Criteria	<15 min	15–30 min	30–60 min	>60 min
time for TP decision, n = 37	–	–	29 (78.4%)	8 (21.6%)
time for TP decision with AI, n = 37	–	6 (16.2%)	28 (75.7%)	3 (8.1%)
3 month, n^3^ = 35				
time for TP decision with AI	1 (2.9%)	13 (37.1%)	21 (60%)	–
6 month, n^6^ = 34				
time for TP decision with AI	3 (8.8%)	20 (58.9%)	11 (32.3%)	–

n – number of DR on study start.

n^3^ = number of DR in 3 months after D and TP decision.

n^6^= number of DR in 6 months after D and TP decision.

Before the introduction of AI, the time to make a decision on the D and TP for all 100% of doctors was more than 30 minutes. At the beginning of the study, 16.2% of doctors managed to establish a D and prescribe a TP in less than 30 minutes, and the percentage of doctors who needed more than 60 minutes to do this decreased by 2.7 times.

In the period from 3 to 6 months, the decision-making time of doctors does not exceed 60 minutes in any case. If at 3 months 40% of doctors have a decision-making time of no more than 30 minutes, then at 6 months this percentage is 67.7%. This indicates the accumulation of experience and the facilitation of work with AI.

The next important question that interested us during the study was the perception of AI by doctors and their impressions (**Table 6**). The survey was conducted at 3 and 6 months, without taking into account their opinions at the start of the study, given the complexity of the initial stage, developing skills for working with AI.

**Table 6:** Assessment AI algorithm by DR, n = 37.

Criteria	0	1	2	3	4
3 month, n^3^ = 35					
overall assessment using AI for D	–	2 (5.7%)	6 (17.1%)	17 (48.6%)	10 (28.6%)
overall assessment using AI for TP	–	1 (2.9%)	5 (14.3%)	23 (65.7%)	6 (17.1%)
convenience AI	1 (2.9%)	1 (2.9%)	14 (40%)	15 (42.8%)	4 (11.4%)
ease of use of AI	–	2 (5.7%)	9 (25.7%)	19 (54.3%)	5 (14.3%)
use AI decrease time for D and TP decision	2 (5.7%)	4 (11.4%)	7 (20%)	16 (45.8%)	6 (17.1%)
can you recommend AI for other colleagues?	–	–	7 (20%)	24 (68.6%)	4 (11.4%)
use AI after end of study	2 (5.7%)	3 (8.6%)	11 (31.4%)	14 (40%)	5 (14.3%)
6 month, n^6^ = 34					
overall assessment using AI for D	–	–	5 (14.7%)	13 (38.2%)	16 (47.1%)
overall assessment using AI for TP	–	–	1 (2.9%)	20 (58.9%)	13 (38.2%)
convenience AI	–	–	2 (5.9%)	28 (82.3%)	4 (11.8%)
ease of use of AI	–	–	1 (2.9%)	14 (41.2%)	19 (55.9%)
use AI decrease time for D and TP decision	1 (2.9%)	4 (11.8%)	9 (26.5%)	14 (41.2%)	6 (17.6%)
can you recommend AI for other colleagues?	–	–	3 (8.8%)	12 (35.3%)	19 (55.9%)
use AI after end of study	–	2 (5.9%)	10 (29.4%)	17 (50%)	5 (14.7%)

n – number of DR on study start.

n^3^ = number of DR in 3 months after D and TP decision.

n^6^= number of DR in 6 months after D and TP decision.

0 – very bad or strictly disagree.

1 - bad or disagree.

2 - moderate or not sure.

3 – good or agree.

4 - very good or strictly agree.

When evaluating the use of AI for D at 3 and 6 months, doctors answered well and very well in 77.2% and 85.3% of cases, respectively. It is more important for the doctor to state the stage of the disease in this case.

Another important factor for the doctor is determining the correct TP. We see that the percentage of good and very good ratings increased from 82.8% at month 3 to 97.1% at month 6.

Convenience and ease of use seem like similar terms, but for doctors, the former indicates more of a psychological and mental perception, while the latter reflects the technical side of use. Convenience with a rating of good or very good increases from 54.2% to 94.1%, and ease of use from 68.6% to 97.1%, indicating the importance of ease and simplicity in using AI.

When asked whether there is a reduction in the time to make a decision on D and TP, we see that agree and strictly agree at month 3 are 62.9% and 58.8% at month 6. This may indicate mastery of AI skills, in contrast to the previous table (**Table 5**), where doctors note an overall reduction in the time to make a decision on D and TP.

The ability to recommend AI to other colleagues is evidence of understanding the importance of its use for D and TP in cancer patients. 80% and 91.2% agree and strictly agree with the absence of any negative response may also indicate the literacy of doctors in terms of AI.

However, the percentage of 54.3% and 64.7% of doctors who want to continue using AI indicates a certain conservatism, since AI is not mandatory for their clinical practice, which involves other diagnosis, complex clinical cases, etc. This indicates the importance of explanatory work, promotion of AI, and involvement in congresses and conferences dedicated to AI and oncology.

## 4 Discussion

### 4.1 MTB – pros and cons

Treatment of cancer patients in comparison with the vast majority of other somatic diseases has significant differences. This is the presence of surgical, therapeutic, immunological, hormonal, targeted, radiation and other treatment methods, which are used both in mono-mode and sequentially or in parallel. In addition, the presence of treatment in the neoadjuvant or adjuvant mode significantly complicates the treatment itself (26).

Therefore, in most specialised medical clinics, MTB has been introduced into the work, the main principle of which is based on the consolidated decision of specialists in various fields involved in the treatment of cancer patients. This has significantly improved the results of setting D and determining TP in patients of various localizations (27, 28).

However, despite the widespread implementation of MTB, today the results of the latter’s activities leave much to be desired. According to the literature, the percentage of change in D and TP remains high (up to 67%). This applies not only to differences between continents, countries, but also to centres within one country (29).

Our results indicate that the percentage of change in D and TP after MTB increases over time and at 6 months is 28.08%, although at 1 month it was 13.12%. In our opinion, the percentage of change in D and TP is high and requires close attention in order to develop measures to reduce it (30–32).

According to the researchers, the main reasons for this are – incomplete or incorrect data in the medical documentation in preparation for the MTB meeting (lack of images, histology, CT/PET data, etc.); different interpretation of images/pathology between specialists; differences in local protocols or resources (regional restrictions in access to therapies/technologies); low quality of evidence for certain clinical situations leads to variability in recommendations; patient preferences or clinical management steps after the MTB meeting that change the implementation of recommendations. Thus, there is a dire need to improve the work of MTB (29, 31).

In our work, we did not set ourselves the task of analysing the reasons leading to the change in D and TP, since our attention is focused only on the efficiency of MTB work.

### 4.2 AI and oncology: ethical, legal, moral, technical, validation, limitation and risk issues

The ethical and legal legitimacy of artificial intelligence in oncology hinges on whether its deployment improves patient-centred outcomes while preserving accountability, transparency, and equity. In this prospective multicentre comparison of multidisciplinary tumour board recommendations against a guideline-based artificial intelligence approach, the central ethical tension is not whether machines should replace clinicians, but whether the decision pathway can be made more reliable, auditable, and consistently aligned with current standards of care without introducing new forms of harm. Contemporary guidance emphasizes that real-world deployment must be governed as a socio-technical intervention: stakeholders define the clinical objective and acceptable trade-offs, the system is evaluated in context, and accountability for decisions remains explicit throughout the lifecycle. A guideline-based system can strengthen moral accountability because it can expose the rule base, the evidence provenance, and the rationale chain linking patient features to recommendations, thereby enabling contestability and structured review. However, this only delivers ethical value if the guideline representation is demonstrably up to date, context-aware (including local access constraints), and paired with human oversight that actively mitigates automation bias (20, 33–46).

From a regulatory perspective, oncology-facing clinical software is increasingly scrutinized for pre-market evidence quality and post-deployment safety monitoring. Analyses of approvals highlight that many medical artificial intelligence products reach practice with limited prospective evaluation and variable external validation, raising concerns about hidden vulnerabilities after implementation (36, 41). This is directly relevant to our study design: prospective, multicentre evaluation is an essential risk-control mechanism, because it surfaces heterogeneity in case-mix, documentation completeness, imaging and pathology interpretation, and resource availability that can systematically shift performance. Dataset shift is not an edge case in oncology; it is a predictable operational reality driven by evolving protocols, changing diagnostics, and population differences, and it can degrade model performance unless monitoring and recalibration are built into governance (42). Therefore, the organizational “operating model” should include performance surveillance, drift detection, and a clear escalation route when discordance emerges between tumour board decisions and guideline-based outputs.

Validation standards must also be aligned with contemporary reporting and evaluation frameworks. For interventional systems, protocol and trial reporting extensions specify how the artificial intelligence component, human-system interaction, error modes, and clinical workflow integration should be documented to enable reproducibility and appraisal. For prediction or risk models often used to triage or prioritise oncology pathways, reporting remains poor across the literature, and high risk of bias is common, undermining clinical readiness. Early-stage decision support evaluations similarly require transparent specification of intended use, clinical context, and human factors, because safety risks frequently arise from interaction failures rather than pure algorithmic error (35). In the present work, a key technical-ethical advantage of guideline-based artificial intelligence is interpretability by construction: it can reduce opaque statistical inference and instead foreground traceable mapping to guideline logic. Nonetheless, it remains vulnerable to mis-specification (for example, incomplete patient data, ambiguous staging descriptors, or discordant pathology inputs) and to guideline limitations, including evidence gaps and slow update cycles. These limitations reintroduce moral questions about fairness and beneficence: consistent decisions are not ethically preferable if they are consistently wrong for under-represented subgroups, complex multimorbidity, or atypical tumour biology (20, 33, 34, 37, 38).

Clinician trust and patient legitimacy are also decisive. Oncologists report that explainability, consent, responsibility allocation, and medico-legal liability are major barriers to adoption, particularly when outputs influence high-stakes treatment selection. This implies that implementation should not treat artificial intelligence as a standalone product, but as a governance-managed capability with explicit communication norms: what the system can and cannot infer, when escalation to tumour board is mandatory, and how disagreements are resolved and documented. Recent oncology reviews converge on the view that the main near-term risk is not speculative “superhuman” behaviour but mundane failure modes: data quality defects, biased training sources, inadequate external validation, workflow mismatch, and uncritical reliance on generated recommendations. In this context, the comparative framing of our study is strategically valuable: discordance between tumour board and guideline-based outputs becomes an auditable signal to priorities root-cause analysis (missing inputs, interpretive disagreement, local resource constraints, or guideline ambiguity) and to design targeted remediation, rather than attributing variability to individual clinician preference (39, 43, 44).

Finally, a forward-looking risk posture requires enterprise-level governance. Practical models proposed within comprehensive cancer centres emphasize structured intake, risk stratification, lifecycle monitoring, and multidisciplinary oversight spanning legal, ethics, adoption, and performance domains (46). Policy-oriented guidance similarly highlights that regulation and institutional controls will continue to evolve, and oncology programmers should plan for adaptive compliance, documentation traceability, and continuous quality assurance rather than one-time “go-live” certification (45). Taken together, the ethical, legal, technical, and validation agenda for our guideline-based artificial intelligence approach is clear: maximize transparency and reproducibility, operationalize prospective multicentre evaluation, embed monitoring for dataset shift and performance drift, and maintain accountable human governance so that clinical responsibility is preserved even as decision processes become more standardized and scalable (20, 33–47).

### 4.3 AI and MTB are both better ways for a clinical decision support system. Is not?

Thus, having considered the role of AI and MTB separately in decision-making regarding D and TP, we see that they have both positive and negative sides. Many researchers have made attempts to combine AI and MTB, but the algorithm and results remain unsatisfactory.

Our approach differs from others in that we have created a decision-making algorithm regarding D and TP, which introduces parallel paths of AI and MTB to the “Concordance” level, after which a decision is made if there is a match, and if there is a mismatch, MTB is re-evaluated.

The implementation of this approach allowed us to reduce TTR by 20.9%, which makes it possible to process a larger number per unit of time and reduce the financial burden.

If the changes in D and TP when making a decision only to use MTB were 13.12% (1 month), 28.08% (6 months); then in the group of patients with a combination of MTB and AI, 4.86% and 14.73%, respectively. The latter indicates that when combining MTB and AI, we managed to obtain good results. This was achieved by reducing the negative aspects of MTB and AI and enhancing the positive ones.

Of course, in our study we included patients with newly diagnosed cancer, without significant comorbidities, and without cancer recurrence. Therefore, our results may differ from other studies of this type.

As for the use of AI by DR who make decisions on D and TP alone, the situation is ambiguous. The results of treating patients with oncological pathology by DR are significantly lower, because they work in non-specialised medical hospitals, they encounter such patients less often, it is more difficult for them to follow guidelines, their theoretical and practical skills need to be improved, etc.

We proposed a DR tool that should significantly help in working with cancer patients, especially for D and TP. Before starting the study, we had doubts, given the certain conservatism of DR in using various models, algorithms, and AI tools, especially in oncology.

If before the implementation of AI in DR work, the time to make a decision was more than 30 minutes in 100% of cases, then after 6 months the time was less than 30 minutes in 67.7% of cases.

After 6 months of using AI, characteristics such as - overall assessment using AI for TP, convenience AI, ease of use of AI, recommendation AI for other colleagues reach more than 90%.

This demonstrates interest and understanding of the use of AI in everyday work with cancer patients. The psychological wariness that exists at the beginning of AI research is changing to pragmatism when DRs see concrete results and effectiveness.

Unlike MTB, a DR does not have colleagues with whom he can exchange ideas. Therefore, evaluating the solutions provided by AI is significantly more difficult and requires the development of appropriate skills.

The literature presents a number of serious problems when working with DR and AI, which cannot always be solved.

### 4.4 Conclusions

Thus, the analysis of the results obtained in the article indicates their certain multidirectionality.

The AI model we proposed, which differs from existing ones in the use of official guidelines, works as an intermediary between patients, guidelines, and LLM, determining the threshold of correct decisions and a high degree of patient de-identification.

Use of MTB and AI combination to optimize TTR, decrease the percentage of change D and TP for 1,6 months, decrease the negative sides of separate MTB and AI. This highlights the positive results both in the early and long term. The study in four oncology centres allowed to expand the geography of patients and reach a larger number of doctors.

Of course, it’s the first step of our work, when we can use this combination for the first detected cases at a basic level. Our patients were without comorbidities. At the same time, a significant part of oncological patients has comorbidities and multiple cancers. In this case, given the increase in the number of tasks, it is necessary to develop an AI agent based on other logistical approaches using orchestration.

Our attempt to help doctors who do not work in specialised institutions was successful. AI cannot replace MTB, but it empowers non-oncological clinicians and DR to generate evidence-based, auditable recommendations in settings with limited specialist capacity. It turned out to be interesting that a certain number of doctors reacted rather “coolly” to the introduction of AI into their work, namely, the lack of desire to use AI after the study was completed, and to recommend the use of AI to other colleagues. This indicates the need for active educational work among all doctors before introducing AI.

The introduction of AI into the work of MTB and other doctors can reduce the financial burden on the healthcare system by establishing the correct diagnosis and choosing the optimal treatment tactics. However, this requires separate research in the future.

## 5 Future trajectory

To reach expert-level autonomy, further work is required a local guideline adaptation, multimodal data ingestion and longitudinal validation across tumour types.

Assessment of the financial efficiency of implementing AI in the work of the MTB.

## Data Availability

The raw data supporting the conclusions of this article will be made available by the authors, without undue reservation.
